# Testing and Analysis of MOSFET-Based Absorber Integrated Antenna for 5G/WiMAX/WLAN Applications

**DOI:** 10.3390/nano12172911

**Published:** 2022-08-24

**Authors:** Elliot O. Omoru, Viranjay M. Srivastava

**Affiliations:** Department of Electronic Engineering, Howard College, University of KwaZulu-Natal, Durban 4041, South Africa

**Keywords:** absorber, antenna, circulator, MOSFET, pulse generator, microelectronics, solid-state electronics

## Abstract

A 3D electromagnetic circuit design and analysis of a MOSFET-based absorber active integrated antenna has been performed. It integrates a transmitting dual-band double material substrate (DMS) cylindrical surrounding patch antenna (CSPA) with a MOSFET-based absorber of reflected radio frequency power. It is a solution to the problem of performance degradation in the power amplifier (PA) resulting from antenna and PA impedance mismatch. This fully integrated MOSFET-based absorber antenna can absorb reflected RF power with a diode-based quasi-circulator as part of the integrated design circuitry. The antenna used for the proposed integrated design will operate at frequencies ranging from 2 GHz to 3 GHz and from 4.6 GHz to 6.1 GHz, thus providing a bandwidth of 1 GHz and 1.5 GHz at a resonance frequency of 2.5 GHz and 5.3 GHz, respectively. This makes it suitable for use in lower and upper bands of WLAN/WiMAX medium RF front-end applications. Furthermore, the condition for MOSFET connected to the absorber (*I_S_* ≤ *I_D_* and *V_DS_* = 0) has been satisfied at both instances of resonance. In this proposed design, an antenna radiation efficiency of 84% has been observed.

## 1. Introduction

The fast expansion of wireless and mobile communication sectors places significant demands on RF front-end systems that are small in size and high in efficiency [1,2,3,4,5]. While trying to meet these demands, there are numerous challenges, especially during the design stage of these systems [6,7]. These challenges have been solved with the introduction of active integrated designs. Active antennas became an intriguing study topic because of advancements in Microwave Integrated Circuit (MIC) technology.

The use of active antenna arrays in mobile communication and beam control has solved the channel capacity limitation problems by increasing the data rate. The advancement of smart antennas is just one of the many advantages of incorporating an active device into a passive antenna. Other benefits include increasing the effective length of short antennas, increasing the bandwidth, improving the noise factor, impedance matching, and increasing receiver antenna sensitivity. One such integrated design is the active integrated antenna (AIA), a novel technology that combines microstrip antennas with active solid-state electronics and microwave components to provide a variety of useful functionalities [8,9,10,11,12]. Integrated antenna design has been introduced to improve the efficiency of front-end systems and exists in recent times in the form of filtering antennas, rectifying antennas, power amplifier antennas, and low noise amplifier (LNA) antennas [13,14,15,16,17].

Compact fifth-mode and eighth-mode balanced substrate integrated waveguide (SIW) band-pass filters for 39 GHz were constructed and evaluated by *Mutepfe* and *Srivastava* [18,19]. A third-order SIW filter/antenna was designed and presented by Lovato et al. [20]; the filter/antenna comprises two cavity resonators, one of which has a slot antenna. The slot antenna maintains the radiation characteristics of the antenna while the excitation of two modes in the slot cavity produces a third-order filtering response. The integrated filter/antenna design had a center frequency of 3.71 GHz, 8.29% fractional bandwidth, and an antenna gain of 5.10 dBi. Huitema et al. [21] presented a miniaturization approach to designing. The initial stage in this concept was to reduce the size of a cavity filter while maintaining a high-quality factor for reduced insertion loss. A second-order filter was used to achieve downsizing by combining a high dielectric permittivity ceramic with a capacitive effect induced by inserting a post in the center of each resonator. Even when the load was greater than 50, the filter performance was maintained.

Additionally, Ji et al. [22] created a ring-type filtering patch antenna that combines a ring patch and a dual-mode microstrip ring resonator. Within the pass-band, two patch modes were created, and two radiation nulls were formed on both sides of the pass-band. This integrated design reduced the overall size and power loss of the front-end system. From the experiment, the max gain of the proposed filtering antenna was above 10 dBi. Wu et al. [23] developed a wideband circularly polarized filtering patch antenna with three minima in the axial ratio response. In that arrangement, the patch antenna was processed in the same manner as the output port and final resonator of the filter network. In a far-field study, the amplitude and phase responses of a circular polarized (CP) wave were mapped to its axial ratio (AR). An 8.8% AR bandwidth was attained on a low profile of 0.028 λ, and the resultant gain demonstrated strong filtering characteristics.

Furthermore, for the operating frequency of 1575.42 MHz of the GPS band, a microstrip antenna with a low noise amplifier was designed by Rusdiyanto et al. [24]. This consists of a single passive antenna and an LNA. According to the simulation, it had a gain of 28.4 dBi; however, the measured gain was 14.77 dBi. The simulation yielded a circular polarization bandwidth of 60 MHz, while the experiment yielded 25 MHz. The operating frequency had a measured return loss of −23.42 dB and impedance bandwidth of 90 MHz. Li et al. [25] created an E-band high-linearity multi-feed antenna LNA front-end with a 45 nm CMOS SOI technology to investigate antenna electronics. This technology supports wireless backhaul and vehicle radar systems. This design takes advantage of antenna noise cancellation and power division to improve the E-band R_X_ Noise Figure (NF) and linearity achievable in silicon front-ends. It was made possible by the high-resistivity silicon substrate employed in it.

Martin et al. [26] designed an LNA and dipole antenna. The LNA has a two-stage cascode based on a SiGe:C HBT with a 130 nm wavelength designed for maximum gain. At *80 GHz*, the circuit is designed for vehicle radar applications. The co-integrated LNA measurement results again reveal NFs of 26 dB and 5 dB, respectively, with a 20 mW power consumption, whereas the antenna exhibits a simulated gain of 0 dB. Using 0.13 m SiGe bipolar complementary metal oxide semiconductor (BiCMOS) technology, a W-band power amplifier (PA) with a dipole antenna was co-designed by Demirel et al. [27]. The co-design enables direct connections between the PA and the transmitter (Tx) antenna, obviating the need for a matching network between the two blocks and allowing for loss reduction, increased energy efficiency, and downsizing. The monolithic integrated circuit (IC) design and measured results were presented. The maximum output power of the 79 GHz differential PA was 17 dBm. With a 1.8 V supply voltage, the chip consumes 300 mA. In the forward direction, the dipole antenna had a gain of −14 dBi, while in the backward direction, it had a gain of −8.5 dBi.

A collaborative design strategy for a co-integrated antenna and PA was provided by Iupikov et al. [28]. This design includes a band-pass RF filter and uses a high-efficiency Doherty PA architecture. After the simulation experiment, a 9% fractional bandwidth (300 MHz) with PAE at the 6 dB output power backed-off level that ranges between 50% and 55% was realized. Similar research work on integrated designs is presented in [29,30,31,32,33]. However, these designed integrated PA antenna structures do not consider the adverse effects of mismatching between the antenna and the PA used in their co-design methodology. One of such effects is arching within the PA structure, which results in PA performance degradation.

In this present research work, a novel MOSFET-based absorbing antenna was designed with a diode-based quasi-circulator as part of its circuitry. This device can be directly connected to the power amplifier without the need for a matching circuit. This design is aimed to reduce the 50 Ω impedance matching limitation on both the PA and the proposed DMS antenna of *Omoru and Srivastava* [34]. On the PA side, the co-design eliminates the challenges of the RF front-end system during matching circuits and eliminates most of the integrated passive parts that serve as a matching circuit. In addition, the rectified reflected power at the MOSFET terminal of the proposed antenna could be amplified and used for low-power operations within the RF front-end system. This paper is organized as follows. Section 2 presents the design of the antenna used for the proposed design. Section 3 shows the design of the MOSFET-based absorber, circulator, and pulse generator used for this antenna. Section 4 presents the design methodology of the antenna. Finally, Section 5 concludes the work and recommends future aspects.

## 2. Double Material Substrate (DMS) Cylindrical Surrounding Patch Antenna (CSPA) Design and Analysis

**Figure 1:**
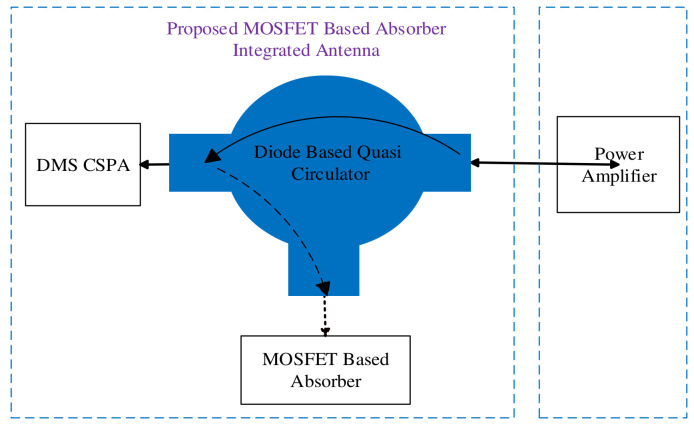
Proposed MOSFET-based absorber active integrated antenna block diagram.

The DMS CSPA was designed using a combination of polyimide substrate and FR-4 substrate with dielectric constants of 3.5 and 4.3, respectively, as in previous work [34]. In continuation of this proposed co-design, the dimensions of the cylindrical patch antenna were altered on purpose by reducing the thickness of the polyimide substrate from 2 mm to 0.5 mm, thus reducing the overall dimension and increasing the bandwidth of the antenna. The revised dimension of the antenna used in the proposed MOSFET-based absorber antenna design is shown in Figure 1 and its parameters are given in Table 1. The adopted modeling, algorithm, and 2D radiation pattern for the cylindrical surrounding patch antenna used in the proposed integrated design have been explained in our previous works [34,35,36].

**Figure 2:**
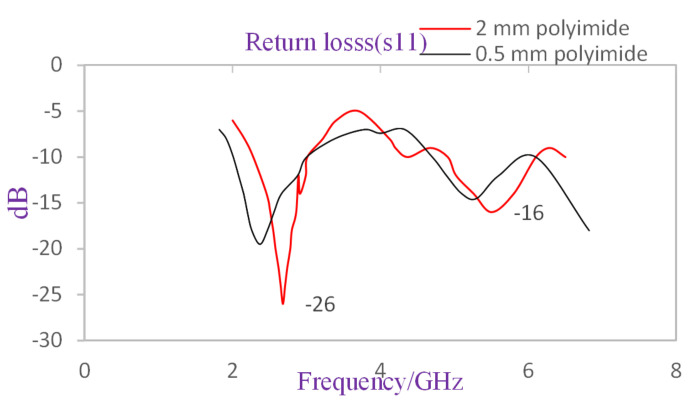
Return loss and equivalent bandwidth at both resonance frequencies using polyimide substrate of thickness 0.5 mm and 2 mm.

As shown in Figure 2, the thinner polyimide substrate caused the lower and upper bands of the WiLAN and WiMax frequency bands to move their backward resonance frequencies from 2.68 GHz to 2.37 GHz and from 5.55 GHz to 5.3 GHz, respectively. In addition, there is an increasing change in return loss from −26 dB to −19.5 dB and from −16 dB to −14.5 dB in the lower and upper bands, respectively. These values may not be practical for the physical implementation of the proposed design, but an increase in return loss values at both resonance frequencies has been observed to be advantageous in terms of providing the needed reflected power for the MOSFET-based absorber in the proposed circuitry.

Before the dimensions of the antenna used for the proposed MOSFET-based absorber active integrated antenna design were altered, a bandwidth of 0.7 GHz (2.28 GHz to 3 GHz) and 1.1 GHz (4.9 GHz to 6.1 GHz) was observed for the lower and upper bands, respectively. Now, using a polyimide value of thickness 0.5 mm resulted in an increased bandwidth of 1 GHz (2 GHz to 3 GHz) and 1.5 GHz (4.6 GHz to 6.1 GHz) for the lower and upper bands, respectively.

**Figure 3:**
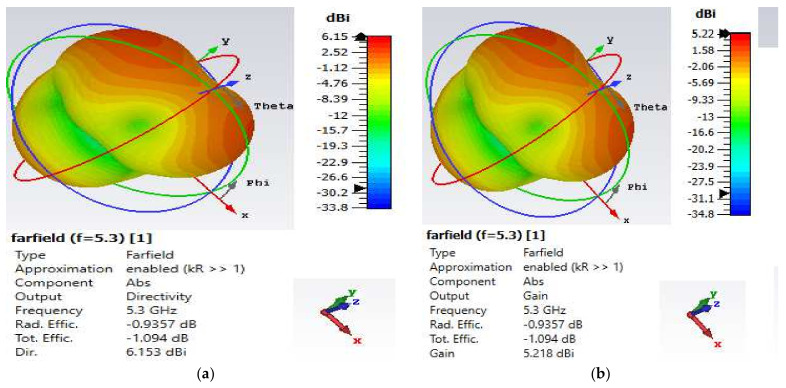
Far-field 3D radiation pattern at 5.3 GHz. (**a**) Directivity value of 6.15 dBi and (**b**) gain value of 5.2 dBi.

**Figure 4:**
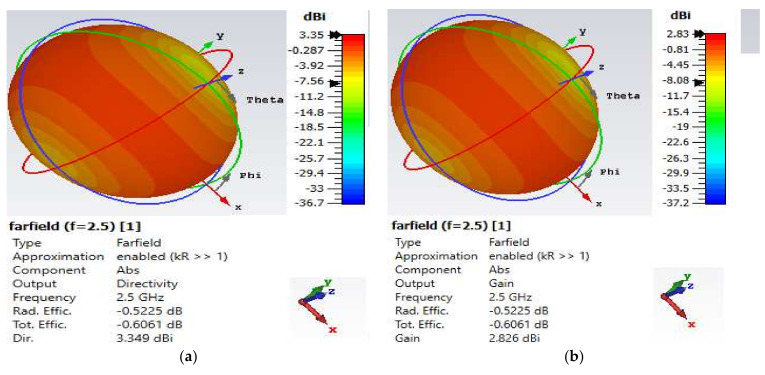
Far-field 3D-radiation pattern at 2.5 GHz. (**a**) Directivity value of 3.35 dBi and (**b**) gain value of 2.83 dBi.

Considering the directivity and gain values presented in Figure 3 and Figure 4, the antenna efficiency factor K and radiation efficiency of the DMS antenna are computed as:

(1) G=KD

(2) $$Radiation\;efficiency=\frac{G}{D}\times 100$$

The dimensionless antenna efficiency factor K must meet the maximum performance requirement of 0 ≤ K ≤ 1, where G and D stand for gain and directivity, respectively [37,38]. A lossless antenna is represented by a value of K = 1. In addition, as shown in Figure 3 and Figure 4, the benefit is never greater than the directivity (D) in practice. Now that G and D values have been input into Equation (1), K has been determined to be equal to 0.843 and 0.848, corresponding to radiation efficiencies of 84.3% and 84.8% for the lower and upper bands, respectively. It has been noted that the computed value of K for the antenna design satisfies the maximum performance criteria of 0 ≤ K ≤ 1. Additionally, the DMS CSPA antenna displayed a radiation efficiency of 81% in both bands prior to changing its dimension.

**Figure 5:**
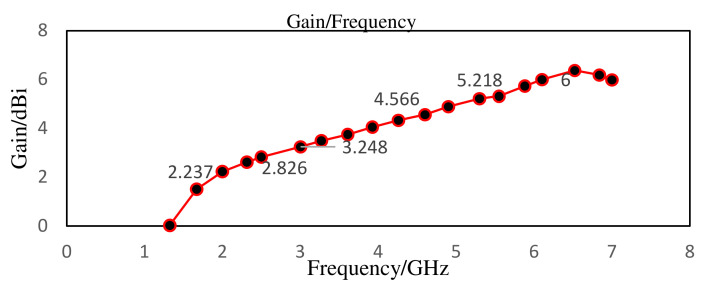
Gain vs frequency plot at both resonance frequencies.

**Figure 6:**
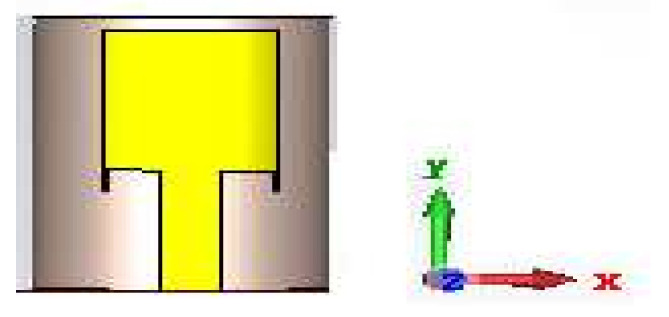
DMS cylindrical patch antenna.

From the gain vs frequency plot presented in Figure 5, it has been observed that there is an increase in gain value as frequency increases from the lowest frequency (2 GHz) of the lower band to the highest frequency (6.1 GHz) of the high band. In essence, this antenna has been excited by the microwave port for testing and experimentation purposes. One of the main benefits of the suggested design is the reasonably small size that results from the combination of FR-4 and polyimide substrate [34,36]. This antenna design, whose front view is depicted in Figure 6, addresses the issue of device fitting and dimension accuracy during manufacture. This advancement will be helpful in the 5G frequency band, where mobile devices now require a smaller antenna to transmit and receive information.

## 3. Design Analysis of SRD Pulse Generator and Diode-Based Quasi-Circulator

To aid the designed model for power amplifier simulation, the SRD pulse generator was used, which provides the required circulator’s incident RF power. In this section, a transient of the pulse generator and a diode-based three-port circulator are presented.

### 3.1. Transient 3D Electromagnetic Circuit Simulation of SRD Pulse Generator

At the prototype design level, there are several techniques for producing wide band pulses [39,40]. Avalanche transistors, tunnel diodes, non-linear transmission lines (NLTLs), photoconductive switches, bipolar transistors, field-effect transistors (FETs), and step recovery diodes (SRDs) are some examples [41]. The pulse generator used in place of the low noise amplifier is made up of a coplanar waveguide layout that includes a helical coil, two lumped capacitors, and a step-recovery diode (SRD). A sinusoidal signal has been used to exit the pulse generator through port-1, thus passing through a low-pass filter (capacitor C_2_ and helical coil) and reaching an SRD connected in parallel with capacitor C_3_. The SRD’s strong nonlinearity produces a very sharp peak, further filtered at the output by a DC blocking capacitor (C_1_), thus producing a signal output at port-2.

**Figure 7:**
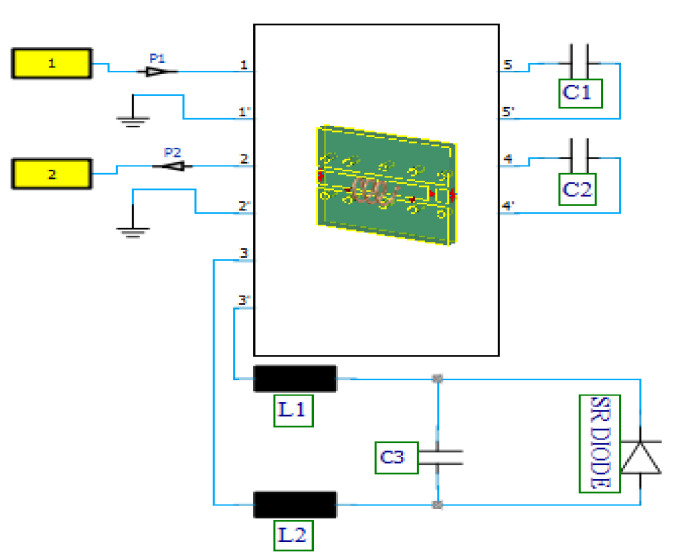
SRD pulse generator used to provide the equivalent incident power for proposed active antenna integrated design testing.

**Figure 8:**
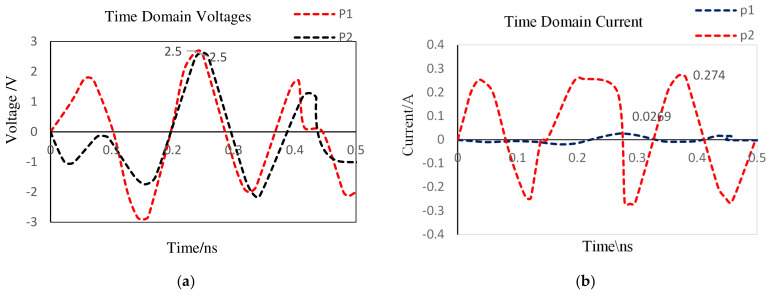
SRD pulse generator time-domain input and output. (**a**) Voltage; (**b**) current.

In Figure 7, the values of capacitors C_1_, C_2_, and C_3_ and inductors L_1_ and L_2_ are 15 pF, 68 pF, 0.35 pF, 0.6 nH, and 0.6 nH, respectively. A maximum frequency of 6 GHz has been defined in both the 3D project and the schematic’s transient tasks to resolve the expected high-frequency material properly. Probes P1 and P2 have been used to record the input and output voltage and current signals at the desired frequency. From Figure 8, the input the peak voltage and current at port-1 or Probe P1 are 2.5 V and 0. 269 A, respectively, signifying a total input power of 0.66 Watts, and values are shown in Table 2.

### 3.2. The 3D Electromagnetic Circuit Simulation of 3-Port Diode-Based Quasi-Circulator Using Directional Couplers

**Figure 9:**
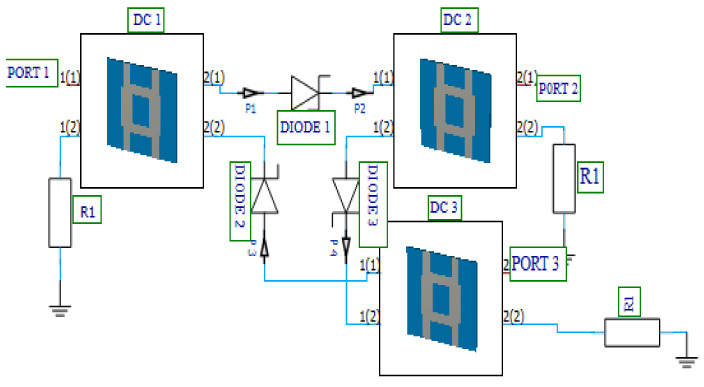
Three-port active diode-based quasi-circulator.

A simplified form of a circulator, a quasi-circulator carries a signal from port-1 to port-2, from port-2 to port-3, and from port-3 to port-1. During this process, one of the ports is isolated; thus, no signal is transmitted to the isolated port. The quasi-circulator has been widely employed as a duplexer in various front-end modules of communication and radar systems, allowing the isolation of different sections within the front-end communication systems [42,43,44,45]. The novel diode-based quasi-circulator model used in the proposed MOSFET-based absorber active integrated antenna was designed with directional couplers, high-frequency Schottky diodes, and resistors. In Figure 9, a schematic depiction of a three-port active diode-based quasi-circulator is shown and detailed using unilateral power divider/combiner and diode characteristics.

Considering the three-port quasi-circulator presented in Figure 8, signals incident on port-1(1) of directional coupler DC1 (port-1 of circulator) are coupled almost in equal shares to the two opposite ports 2(1) and 2(2). At this stage, diode 1 (DIODE 1) is ON while diode 2 (DIODE 2) is OFF. Since DIODE 2 is OFF, signals incident on port-1(1) of directional coupler DC1 will be allowed exit port-2(1), flowing through DIODE 1 to port-1(1) of directional coupler DC2. When the signals reach port-1(1) of directional coupler DC2, the signals are also coupled in almost equal shares to the two opposite ports, ports 2(1) and 2(2). In port-2(2), the signal is matched to a 50 Ω resistor R_1_, thus allowing the signal to flow through port-2(1) of directional coupler DC2 (port-2 of the circulator). This configuration isolates port-3 of the circulator. A similar process occurs when the signal enters at ports-2 and -3, and then we have an active circulator, as shown in Figure 9.

**Figure 10:**
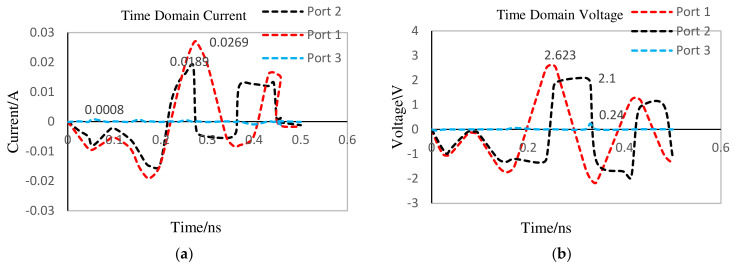
Diode-based quasi-circulator port. (**a**) Current and (**b**) voltage.

To test the functionality of the designed diode-based quasi-circulator in terms of power routing capability, the output power from port-2 of the pulse generator presented in Section 3.1 is connected to port-1 of the designed circulator, and peak current and voltage values at each port of the circulator are shown in Figure 10a,b, respectively. Table 3 presents each port’s current, voltage, and power values. It has been observed that more than half of the power incident on port-1 of the circulator is transferred to port-2. Moreover, port-3 is almost fully isolated with an output power of 192 mW, which is minimal compared to the power incident on port-1.

## 4. Electromagnetic Circuit Simulation Analysis of MOSFET-Based Absorber Integrated Antenna

The absorbers are classified as broadband absorbers or narrowband absorbers [46,47]. Broadband absorbers work across a large frequency range, but typically exhibit low levels of attenuation, whereas narrowband absorbers work well over a smaller frequency range but exhibit high levels of attenuation [48]. Hu et al. [49] developed a novel downsized absorber frequency selective surface for low-frequency transmission/high-frequency absorption. In this instance, the lower layer’s selection zone of the symmetrical frequency was below 1.8 GHz, and the wave transmission rate was more than 84%. A wave-absorbing frequency selective surface (AFSS) with low-frequency transmission and high-frequency absorption was proposed by Han et al. [50]. To prevent the frequency selection feature from affecting the microwave absorption effect, the unit uses a method that loads the frequency selection structure proportionally to the absorber’s reflective metal surface.

The suggested model offered more than 10 dB insertion loss between 4 GHz and 5.5 GHz. Saikia et al. [51] designed and optimized an absorber design for the C-band using equivalent circuit analysis. Capacitive and analog circuit layers with lumped resistors make up the design. The entire wave simulation of the constructed microwave absorber displays a bandwidth of below −10 dB absorption from 4.23 GHz to 8.13 GHz. Although most absorbers are foam absorbers, depending on the frequency needed and the level of absorption they can deliver, they could also be analog circuits [52].

**Figure 11:**
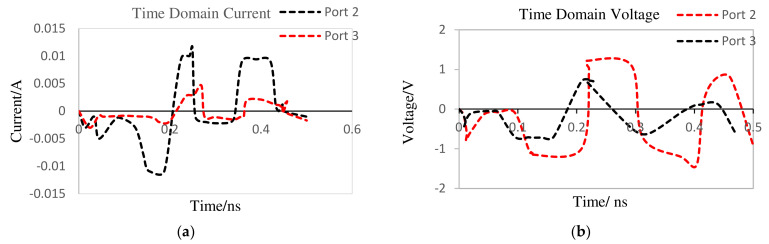
Circulator port after antenna attachment. (**a**) Current and (**b**) voltage.

For a MOSFET-based absorber design, it is vital to know the amount of predicted reflected power from the antenna branch of the proposed MOSFET-based antenna design. Knowledge of the peak and RMS values of current, voltage, and power will enable engineers to analyze the proper values of resistors, capacitors, and inductors (resistor inductor–capacitor filter) for the MOSFET-based absorber design [53]. To compute these values, port-1 of the circulator presented in Section 2 is connected to the SRD pulse generator’s output, and port-2 of the circulator is connected to the DMS dual-band antenna. After carrying out a transient 3D EM/co-simulation of the connected device, the reflected peak current and peak voltage from the antenna branch are observed at port-3, with values of 4.36 mA and 722 mV, respectively, as shown in Figure 11a,b.

Equations (3) through (12) have been adopted from the existing literature [3,53,54,55,56,57]:

(3) $$V_{rms}={\;V}_{peak}\times 0.707$$

(4) $$V_{DC}=\frac{2V_{peak}}{\pi }$$

(5) $$I_{rms}=0.707\times I_{peak}$$

(6) $$I_{DC}=\frac{2I_{peak}}{\pi }$$

Using Equations (3) and (6), the rms and DC values of reflected current and voltage signals have been computed as *V_rms_* (495 mV), *I_rms_* (3.08 mA), *V_dc_* (446 mV), and *I_dc_* (2.8 mA). In addition, the ripple factor and impedance of the inductor (*X_L_*) and capacitor (*X_C_*) influence the capacitor and inductor values required for the MOSFET absorber design. The ripple factor, the impedance of the inductor, and the impedance of the capacitor are defined as:

(7) $$RippleFactor=\frac{\sqrt{I_{rms}^{2}-I_{DC}^{2}}}{I_{DC}}$$

(8) $$X_{L}=\frac{V_{peak}}{I_{rms}}$$

(9) $$X_{C}=\sqrt{\frac{3X_{L}\times Ripple\;factor}{\sqrt{2}}}$$

The ripple factor increases to 0.46 when these values for *I_rms_* and *I_DC_* are substituted into Equation (7). Additionally, 227 Ω and 14 Ω were calculated as the respective impedances of the inductor and capacitor. Regarding the design frequency, 6 GHz was chosen for the MOSFET-based absorber. Values of inductors and capacitors for the MOSFET-based absorber can be computed as:

(10) $$L=\frac{X_{L}}{2\pi f}$$

(11) $$C=\frac{1}{2\pi fX_{C}}$$

The values of the inductor (*L*) and capacitor (C_4_ and C_5_) were calculated as 8 nH and 2.5 pF, respectively. The load resistor (*R_2_*) used in the MOSFET-based absorber was computed as a function of the voltage and current component of the peak reflected power, as shown in Equation (12), and the value is 157 kΩ.

(12) $$R=\frac{V_{Peak}}{I_{Peak}}$$

**Figure 12:**
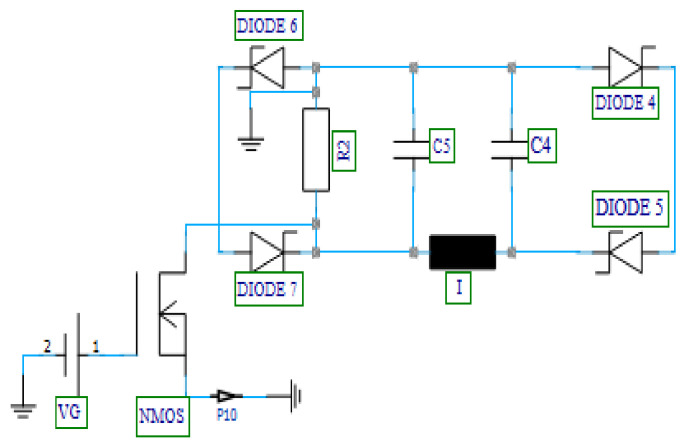
Designed MOSFET-based absorber.

The MOSFET-based absorber schematic is presented in Figure 12. In order to complete the rectification process while taking into account the ON and OFF states of the diodes and the potentials at each node of the rectifier, it has been demonstrated that the absorber is a combination of an RLC filter, an N-channel MOSFET, and a four-diode combination [55,56,57]. The function of the RLC filter, in this case, is to reduce the pulses at the output of the rectifier; this allows for maximum performance of the MOSFET device in the absorber. To turn ON the MOSFET, a DC voltage (V_G_) of 12 V is applied to the MOSFET gate, thus allowing the flow of the current from the drain to the source.

**Figure 13:**
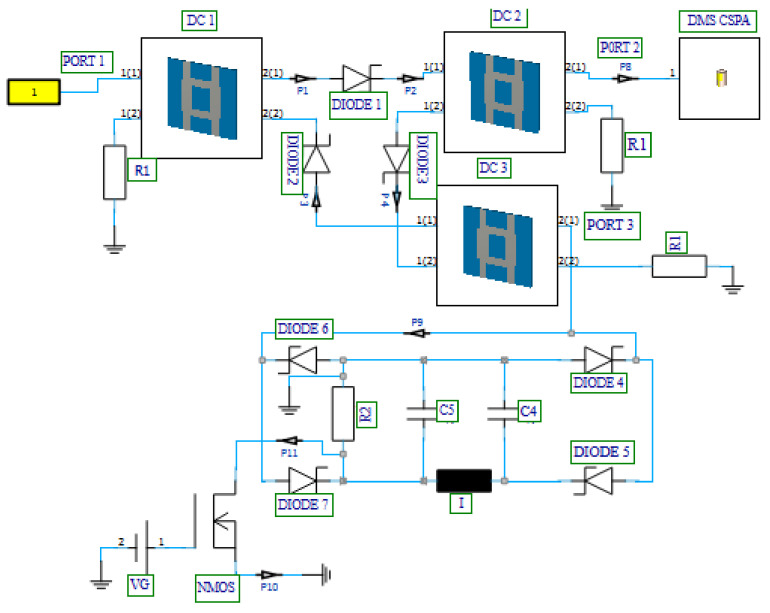
Proposed MOSFET-based absorber integrated antenna.

Figure 13 shows the 3D EM/circuit co-simulation schematic of the integrated SiO_2_ MOSFET-based absorber and dual-band double-material substrate CSPA. To run the co-simulated schematic, the output of the pulse generator was connected to port-1 of the presented schematic (Figure 13). The reflected power resulting from the mismatch between the pulse generator (PA) and the DMS CSPA is rectified, filtered, and observed at P11 (drain terminal of the MOSFET) and P10 (source terminal of the MOSFET). In the proposed MOSFET-based absorber integrated antenna model, reflection is bound to occur from the antenna branch (port-2) under two MOSFET source current (I_S_) operation conditions. The condition for MOSFET absorption has been used from Refs. [53,54,55,56].

**Figure 14:**
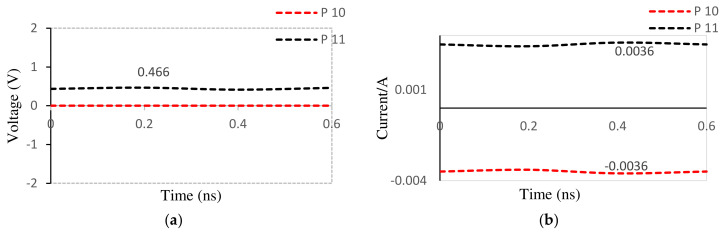
Drain/source (**a**) voltage and (**b**) current.

After running a transient simulation on the presented schematic of the MOSFET-based absorber antenna, the drain voltage and current were observed as in Figure 14. Considering the proposed antenna, the MOSFET-based absorber serves its purpose of absorbing the reflected signal from the antenna branch of the circulator, if the relationships *I_S_* ≤ *I_D_* (current at P10 greater than or equal to current at p11) and *V_DS_ =* 0 (voltage at p10 equal to 0) are justified. When these conditions are satisfied, adverse effects (such as aching within the transmission line or signal route within the circulator) are eliminated from a mismatch between the PA and the antenna.

From Table 4, it is observed that the condition for MOSFET absorption is satisfied for the designed antenna (source voltage is 0 and the source current is negative compared to the drain current, implying a condition of *I_S_* ≤ *I_D_*).

## 5. Conclusion and Future Recommendations

The 3D EM/circuit co-simulation of the MOSFET-based absorber integrated antenna has been observed to have a suitable reflection absorbing capability by satisfying the condition for MOSFET absorption. This device, when directly connected to the power amplifier’s output, reduces the effect of impedance mismatch between the PA and the antenna because the reflected power that would have resulted in arching within the PA structure is absorbed by the MOSFET absorber in the antenna construct. Another advantage of this design is that the model can function as a rectifying antenna for energy harvesting. Furthermore, the dual material antenna used in the proposed integrated design was designed to resonate at 2.5 GHz (2 GHz~3 GHz) and 5.3 GHz (4.6 GHz~6.1 GHz), hence making it suitable for the lower and upper bands of WLAN and WiMAX and the middle band of 5G technology (2.3 GHz to 2.4 GHz and 2.5 GHz to 2.69 GHz). This antenna design can be used in the front end of communication systems, signal intelligence systems, and other forms of transportation (intelligence, surveillance, and reconnaissance) systems.

As a continuation of this work, we will present a block-by-block performance analysis of the proposed model to validate the usability of the 3D electromagnetic circuit simulation design in the 5G regime. Thereafter, a fabricated device will be realized.

## Figures and Tables

**Table 1:** Parameters used for CSPA modeling.

Description	Parameter	Value (in mm)
The outer radius of ground	*R_go_*	12.12
Inner radius of ground	*R_gi_*	11.32
Outer radius of the polyimide substrate	*R_so.pl_*	12.62
Outer radius of FR-4 substrate	*R_so-fr-4_*	15.42
Length of ground	*L_g_*	22.87
Length of substrate	*L_s_*	39.84
Substrate and patch top difference	*∆L*	2.070
Inset feed length	*Yo*	3.11
Width of feed	*W_f_*	5.70
Height of patch	*H_p_*	0.8
Length of patch	*L_p_*	11.04
Width of patch	*W_p_*	17.26
Assumed width of the ground	*W_g_*	46.06
Thickness of ground	*H_g_*	0.8
Thickness of polyimide substrate	*H_s.pl_*	2.0
Thickness of FR-4 substrate	*H_s.fr-4_*	2.8
Effective length	*L_eff_*	15.27

**Table 2:** Equivalent voltage, current, and power at the input and output terminal of the pulse generator.

	Voltage	Current	Power
P1	2500 mV	263 mA	660 mW
P2	2500 mV	26.9 mA	67 mW

**Table 3:** Circulator port parameters.

Ports	Current (mA)	Voltage (mV)	Power (mW)
1	26.9	2623	70,559
2	18.9	2100	39,690
3	0.8	240	192

**Table 4:** MOSFET drain and source terminal parameters.

Terminal	Voltage (mV)	Current (mA)
Source (P11)	0	−3.6
Drain(P10)	466	3.6

## References

[1] Fazel Z., Atarodi M., Sadughi S. (2021). A system-level design method for RF receiver front-end with low power consumption. Analog Integr. Circuits Signal Process..

[2] Wang D., Chen L., Gao L. Simulation Study on the Influence of the Damage of RF Front-end Limiter to the Performance of Satellite Communication System. Proceedings of the 2020 6th Global Electromagnetic Compatibility Conference (GEMCCON).

[3] Srivastava V.M., Singh G. (2014). MOSFET Technologies for Double-Pole Four Throw Radio Frequency Switch.

[4] Emara M.K., King D.J., Nguyen H.V., Abielmona S., Gupta S. (2020). Millimeter-Wave Slot Array Antenna Front-End for Amplitude-Only Direction Finding. IEEE Trans. Antennas Propag..

[5] Duplouy J., Morlaas C., Aubert H., Potier P., Pouliguen P. (2019). Wideband Vector Antenna for Dual-Polarized and Three-Dimensional Direction-Finding Applications. IEEE Antennas Wirel. Propag. Lett..

[6] Al-Hiti A.S. (2019). Design of rectangular microstrip patch antenna for WLAN and WiMAX. ARPN J. Eng. Appl. Sci..

[7] Serghiou D., Khalily M., Singh V., Araghi A., Tafazolli R. (2020). Sub-6 GHz Dual-Band 8 × 8 MIMO Antenna for 5G Smartphones. IEEE Antennas Wirel. Propag. Lett..

[8] Valizade A., Rezaei P., Orouji A.A. (2014). A new design of dual-port active integrated antenna for 2.4/5.2 GHz WLAN Applications. Prog. Electromagn. Res. B.

[9] Chang K., York R.A., Hall P.S., Itoh T. (2002). Active integrated antennas. IEEE Trans. Antennas Wirel. Propag..

[10] Aghazadeh S.R., Martinez-Garcia H., Barajas-Ojeda E., Saberkari A. (2022). 3–5-GHz, 385–540-ps CMOS true-time delay element for ultra-wideband antenna arrays. AEU Int. J. Electron. Commun..

[11] Kumari R., Basu A., Koul A.S.K. (2021). Frequency Reconfigurable High Power Gan/Algan Hemt Based Self Oscillating Active Integrated Antenna. Prog. Electromagn. Res. Lett..

[12] Singh D., Srivastava V.M. (2018). An analysis of RCS for dual-band slotted patch antenna with a thin dielectric using shorted stubs metamaterial absorber. AEU Int. J. Electron. Commun..

[13] Hedayati M.K., Abdipour A., Shirazi R.S., Ammann M.J., John M., Cetintepe C., Staszewski R.B. (2019). Challenges in On-Chip Antenna Design and Integration with RF Receiver Front-End Circuitry in Nanoscale CMOS for 5G Communication Systems. IEEE Access.

[14] Hara S., Suzuki A., Hirayama H. Proposal and demonstration of power conversion-chip/amplifier integrated antenna. Proceedings of the 50th European Microwave Conference.

[15] Singh N., Kanaujia B.K., Beg M.T., Kumar S., Khandelwal M.K. (2018). A dual band rectifying antenna for RF energy harvesting. J. Comput. Electron..

[16] Yang S.J., Pan Y.M., Shi L.-Y., Zhang X.Y. (2020). Millimeter-Wave Dual-Polarized Filtering Antenna for 5G Application. IEEE Trans. Antennas Propag..

[17] Tang M.-C., Wen Z., Wang H., Li M., Ziolkowski R.W. (2017). Compact, Frequency-Reconfigurable Filtenna with Sharply Defined Wideband and Continuously Tunable Narrowband States. IEEE Trans. Antennas Propag..

[18] Mutepfe C.D.K., Srivastava V.M. (2021). Design and Analysis of Compact 5th Mode Balanced Substrate Integrated Waveguide Band-Pass Filter for 39 GHz. Int. J. Commun. Antenna Propag..

[19] Mutepfe C.D.K., Srivastava V.M. (2021). Designing of Novel Eighth-Mode Forth-Order Substrate Integrated Waveguide Band-Pass Filter with High Selectivity. J. Commun..

[20] Lovato R., Gong X. (2018). A Third-Order SIW-Integrated Filter/Antenna Using Two Resonant Cavities. IEEE Antennas Wirel. Propag. Lett..

[21] Huitema L., Dia Y., Thevenot M., Bila S., Perigaud A., Delaveaud C. (2021). Miniaturization of a Filter-Antenna Device by Co-Design. IEEE Open J. Antennas Propag..

[22] Ji S., Dong Y., Fan Y. Co-designed Ring-Type Filtering Patch Antenna with Dual-mode Microstrip Ring Resonator. Proceedings of the 2020 International Conference on Microwave and Millimeter Wave Technology (ICMMT).

[23] Wu Q.-S., Zhang X., Zhu L. (2018). Co-Design of a Wideband Circularly Polarized Filtering Patch Antenna with Three Minima in Axial Ratio Response. IEEE Trans. Antennas Propag..

[24] Rusdiyanto D., Zulkifli F.Y. Antenna Integrated with Low Noise Amplifier Operating at L1 GPS Application. Proceedings of the 2019 IEEE Asia-Pacific Microwave Conference (APMC).

[25] Li S., Chi T., Jung D., Huang T.Y., Huang M.Y., Wang H. An E-Band high-linearity antenna-LNA front-end with 4.8 dB NF and 2.2 dBm IIP3 exploiting multi-feed on-antenna noise-canceling and gm-boosting. Proceedings of the 2020 IEEE International Solid-State Circuits Conference-(ISSCC).

[26] Martin N., Taris T., Bégueret J.-B., Person C., Belot D. 80 GHz co-designed LNA and antenna for automotive radar. Proceedings of the 21st IEEE International Conference on Electronics, Circuits and Systems (ICECS).

[27] Demirel N., Pinto Y., Calvez C., Titz D., Luxey C., Person C., Gloria D., Belot D., Pache D., Kerhervé E. (2013). Codesign of a PA–antenna block in silicon technology for 80-GHz radar application. IEEE Trans. Circuits Syst. II Express Briefs.

[28] Iupikov O.A., Perez-Cisneros J.R., Meyer P., Åkesson D., Maaskant R., Buisman K., Rehammar R., Fager C., Marianna V., Ivashina M.V. (2021). Cavity-backed patch antenna with distributed multi-port feeding, enabling efficient integration with Doherty power amplifier and band-pass filter. IEEE Trans. Antennas Propag..

[29] Hu Y.-Y., Sun S., Xu H. (2020). Compact Collinear Quasi-Yagi Antenna Array for Wireless Energy Harvesting. IEEE Access.

[30] Wang M., Fan Y., Yang L., Li Y., Feng J., Shi Y. (2019). Compact dual-band rectenna for RF energy harvest based on a tree-like antenna. IET Microw. Antennas Propag..

[31] Zhang B., Xue Q. (2018). Filtering Antenna with High Selectivity Using Multiple Coupling Paths from Source/Load to Resonators. IEEE Trans. Antennas Propag..

[32] Li Y., Zhao Z., Tang Z., Yin Y. (2020). Differentially Fed, Dual-Band Dual-Polarized Filtering Antenna with High Selectivity for 5G Sub-6 GHz Base Station Applications. IEEE Trans. Antennas Propag..

[33] Roy G.M., Dwari S., Kanaujia B.K., Kumar S., Song H. (2021). Active feedback supported CMOS LNA blended with coplanar waveguide-fed antenna for Wi-Fi networks. IET Microw. Antennas Propag..

[34] Omoru E., Srivastava V.M. (2021). Comparative analysis of dual-band double-material substrate based cylindrical surrounding patch antenna for 5G/WiMAX/WLAN applications. Int. J. Commun. Antenna Propag..

[35] Maduagwu U.A., Srivastava V.M. (2021). Sensitivity of Lightly and Heavily Dopped Cylindrical Surrounding Double-Gate (CSDG) MOSFET to Process Variation. IEEE Access.

[36] Omoru E.O., Srivastava V.M. (2022). Radiation pattern, surface current distribution, Q-factor improvement technique using DMS CSPA: Part-II. J. Commun..

[37] Umayah E.N., Srivastava V.M. (2020). Comparative analysis of feeding techniques for cylindrical surrounding patch antenna. Int. J. Electr. Comput. Eng..

[38] Constantine A. (2016). Balanis, Antenna Theory: Analysis and Design.

[39] Zeng F., Yao J. (2006). An approach to ultrawideband pulse generation and distribution over optical fiber. IEEE Photon- Technol. Lett..

[40] Rahman M., Wu K. (2022). A reconfigurable picosecond pulse generator in non-linear transmission line for impulse radar ultra-wideband applications. IEEE Microw. Wirel. Compon. Lett..

[41] Xu W., Chen J., Shi Z., Zhang A. A Novel Picosecond-Pulse Circuit Based on Marx Structure and SRD. Proceedings of the 4th International Conference on Electronic Information and Communication Technology (ICEICT).

[42] Wang S., Lee C.H., Wu Y.B. (2016). Fully integrated 10-GHz active circulator and quasi-circulator using bridged-T networks in standard CMOS. IEEE Tran. Very Large Scale Integr. Syst..

[43] Chang C.H., Lo Y.T., Kiang J.F. (2010). A 30 GHz active quasi-circulator with current-reuse technique in 0.18 µm CMOS technology. IEEE Microw. Wirel. Compon. Lett..

[44] Porranzl M., Wagner C., Jaeger H., Stelzer A. (2015). An Active Quasi-Circulator for 77 GHz Automotive FMCW Radar Systems in SiGe Technology. IEEE Microw. Wirel. Components Lett..

[45] Yoo B.Y., Park J.H., Yang J.R. (2018). Quasi-Circulator Using an Asymmetric Coupler for Tx Leakage Cancellation. Electronics.

[46] Houtz D.A., Gu D. (2018). A Measurement Technique for Infrared Emissivity of Epoxy-Based Microwave Absorbing Materials. IEEE Geosci. Remote Sens. Lett..

[47] Xu Q., Huang Y. (2018). Anechoic and Reverberation Chambers: Theory, Design and Measurements.

[48] Luo G.Q., Yu W., Yu Y., Zhang X.H., Shen Z. (2020). A Three-Dimensional Design of Ultra-Wideband Microwave Absorbers. IEEE Trans. Microw. Theory Tech..

[49] Hu D., Zhai H., Li S., Xiong W., Zhang L. A New Miniaturized Absorber Frequency Selective Surface for Low Frequency Wave Transmission and High Frequency Absorption. Proceedings of the 2019 International Conference on Microwave and Millimeter Wave Technology (ICMMT).

[50] Han Y.D., Zhu B.C., Yan Z.H. Design of a wave-absorbing frequency selective surface unit. Proceedings of the 2018 Cross Strait Quad-Regional Radio Science and Wireless Technology Conference (CSQRWC).

[51] Saikia M., Srivastava K.V. Design of Thin Broadband Microwave Absorber using Combination of Capacitive and Circuit Analog Absorbers. Proceedings of the 2018 IEEE Indian Conference on Antennas and Propogation (InCAP).

[52] Zhang Q., Shen Z., Wang J., Lee K.S. (2012). Design of a switchable microwave absorber. IEEE Antennas Wirel. Propag. Lett..

[53] Sedra A.S., Smith K.C. (2014). Microelectronic Circuits: Theory and Applications.

[54] Boylestad R.L., Nashelsky L. (2012). Electronic Devices and Circuit Theory.

[55] Uchechukwu M.A., Srivastava V.M. (2020). Channel length scaling pattern for Cylindrical Surrounding Double-Gate (CSDG) MOSFET. IEEE Access.

[56] Gowthaman N., Srivastava V.M. (2021). Parametric analysis of CSDG MOSFET with La_2_O_3_ gate oxide: Based on electrical field estimation. IEEE Access.

[57] Rashid M.H. (2018). Power Electronics Handbook: Devices, Circuits and Applications.

